# Genome-wide identification and integrated analysis of lncRNAs in rice backcross introgression lines (BC_2_F_12_)

**DOI:** 10.1186/s12870-020-02508-y

**Published:** 2020-06-29

**Authors:** Mengdi Li, Aqin Cao, Ruihua Wang, Zeyu Li, Shaoqing Li, Jianbo Wang

**Affiliations:** State Key Laboratory of Hybrid Rice, College of Life Sciences, Wuhan University, Wuhan, 430072 China

**Keywords:** Long non-coding RNA, Backcross introgression lines, Progenies, Transcriptome sequencing, Rice

## Abstract

**Background:**

Distant hybridization is an important way to create interspecific genetic variation and breed new varieties in rice. A lot of backcross introgression lines (BILs) had been constructed for the scientific issues in rice. However, studies on the critical regulatory factor lncRNA in cultivated rice, wild rice and their BIL progenies were poorly reported.

**Results:**

Here, high-throughput RNA sequencing technology was used to explore the functional characteristics and differences of lncRNAs in *O. sativa*, *O. longistaminata* and their three BC_2_F_12_ progenies. A total of 1254 lncRNAs were screened out, and the number of differentially expressed lncRNAs between progenies and *O. sativa* were significantly less than that between progenies and *O. longistaminata*. Some lncRNAs regulated more than one mRNA, and 89.5% of lncRNAs regulated the expression of target genes through *cis*-acting. A total of 78 lncRNAs and 271 mRNAs were targeted by 280 miRNAs, and 22 lncRNAs were predicted to be the precursor of 20 microRNAs. Some miRNAs were found to target their own potential precursor lncRNAs. Over 50% of lncRNAs showed parental expression level dominance (ELD) in all three progenies, and most lncRNAs showed ELD*-O. sativa* rather than ELD-*O. longistaminata*. Further analysis showed that lncRNAs might regulate the expression of plant hormone-related genes and the adaptability of *O. sativa*, *O. longistaminata* and their progenies.

**Conclusions:**

Taken together, the above results provided valuable clues for elucidating the functional features and expression differences of lncRNAs between *O. sativa*, *O. longistaminata* and their BIL progenies, and expanded our understanding about the biological functions of lncRNAs in rice.

## Background

Rice (*Oryza sativa* L.) is one of the staple foods for world population, and its yield is crucial for global food production. With the advantages of moderate genome size and high-quality reference genome, rice is widely regarded as a typical model plant to study the genetic structure and function of monocotyledons. Genus *Oryza* has evolved into 24 species, consisting of 2 cultivated species (*O. sativa* and *O. glaberrima*) and 22 wild species [1, 2]. Cultivated rice has lost many important useful genes after a long period of artificial selection. However, wild rice, which has experienced harsh natural environment, contains a large number of valuable genes and is a valuable resource in rice breeding [3]. Interspecific distant hybridization between different species with excellent genes and distant genetic relationships is an important way to create genetic variation and breed new varieties, which is also an important driver of genome evolution and speciation [4]. A generally accepted effective strategy to expand the genetic diversity of cultivated rice is to identify and utilize valuable alleles with agronomic traits from wild rice and introduce them into cultivated rice by crossbreeding backcross [5, 6]. In the past few decades, a lot of backcross introgression lines (BILs) had been constructed to study scientific issues in rice, such as drought resistance [7, 8] genomic structure [3], hybrid sterility [5] and gene, miRNA and metabolic profiling [2, 9]. *O. longistaminata* is a perennial wild rice widely distributed in tropical Africa with strong resistance to biotic and abiotic stresses, strong rhizomes, long anthers and self-incompatibility [10–12].

Long non-coding RNA (lncRNA) refers to RNA with a length of more than 200 bp and no protein coding capacity. LncRNAs are involved in a variety of molecular and genetic mechanisms, including transcriptional level, post-transcriptional level and epigenetic level [13, 14]. LncRNAs are involved in many biological processes, including growth of human tumor cells, plant morphogenesis, biotic stress and abiotic threats [15–17]. The functions of lncRNAs are divided into four categories, including signal, decoy, guide, and scaffold [18]. Specifically, lncRNAs could regulate spatial/temporal expression of genes when they act as signal molecules [19]. LncRNAs could participate in maintaining the stability of gene expression by acting as decoys or target mimics of miRNAs [20–22]. LncRNAs could also guide the ribonucleoprotein complex to locate at a specific site to play their guiding role [23]. In addition, lncRNAs could be used as scaffolds to form skeletal complexes with transcription factors, which can regulate the up−/down-stream effector elements and further activate or inhibit the transcription of genes [24]. Moreover, lncRNAs could regulate the expression of protein-coding genes at the transcriptional level through *cis*-regulation or *trans*-regulation. When lncRNA *cis*-regulates the target gene, it is encoded from the nucleic acid chain in which their target gene was located. On the contrary, the nucleic acid sequence encoding lncRNA was not on the same nucleic acid chain as the target gene coding sequence when lncRNA *trans*-regulate the target gene. For instance, a lncRNA (COLDAIR) *cis*-regulated the *FLOWERING LOCUS C* (*FLC*) gene, which was important in the regulation of flowering time in Arabidopsis; COLDAIR also *trans*-regulated the *FLC* gene by binding to protein complex PcG [25, 26]. In addition, lncRNAs could be used as precursors of microRNAs (miRNAs), and some lncRNAs could also bind to miRNAs directly to regulate their functions [27]. Although a large number of lncRNAs has been identified in previous studies, the research on their biological functions is still in the initial stage, especially in plants.

Recently, high-throughput sequencing technology were often used to detect low-level expressed transcripts and identify numerous mRNAs, small RNAs and lncRNAs with important roles in biological processes [15, 16, 28, 29]. In our previous study, the expression patterns of genes and miRNAs in *O. sativa*, *O. longistaminata* and their three BIL progenies were performed, and the regulation of miRNAs on genes were also explored [2]. In this study, the high-throughput strand-specific RNA sequencing (ss-RNAseq) technology was used to study the expression differences and characteristics of lncRNAs and their target genes in *O. sativa*, *O. longistaminata* and their three BILs (BC_2_F_12_) progenies (L1710, L1817 and L1730). The lncRNAs acting as precursors or target mimics of miRNAs were also studied in these species. Further analysis showed that parental expression level dominance (ELD) phenomenon was the most common event in the three progenies. This work could provide valuable clues to reveal the molecular mechanisms of gene introgression of wild rice through hybrid and backcross.

## Results

### Overview of the sequencing data

To explore the expression characteristics of lncRNAs and their roles in *O. sativa*, *O. longistaminata* and their three BILs progenies at jointing-booting stage, the strand-specific RNA-seq (ssRNA-seq) technology was used in this study. Overall, an average of 13.70 Gb data with each sample was obtained and the gene expression correlations among three biological replicates were high, with average coefficient (*R*^*2*^) of 0.98 (Supplementary Fig. S1). On average, 101,808,785 (L1710), 91,859,276 (L1817), 88,566,772 (L1730), 98,879,278 (*O. sativa*) and 103,545,393 (*O. longistaminata*) raw reads were generated respectively, of which more than 94% reads were clean reads (Supplementary Table S1). All clean reads obtained from sequencing of 15 ssRNA libraries were uploaded to the NCBI’s Sequence Read Archive (SRA) database (https://trace.ncbi.nlm.nih.gov/Traces/sra/sra.cgi?view=announcement) with accession numbers SRR9822767-SRR9822781. After re-assembling and mapping, around 56% clean reads in *O. longistaminata* and 70% clean reads in three progenies and *O. sativa* were uniquely mapped to the rice reference genome (Supplementary Table S1) and 66,338 transcripts were identified as known mRNAs. Moreover, known mRNAs and transcripts whose information cannot be recognized were eliminated, and the remaining transcripts (described as novel transcripts in the following text) were further identified as candidates for lncRNAs. As a result, 16,038 novel transcripts were assembled, most of which were within 4500 nt in length, and transcripts containing more than 10 exons accounted for a high proportion, while nearly half of the genes had only one transcript (Supplementary Table S2).

### Identification and the sequence characteristics of lncRNAs

**Fig. 1:**
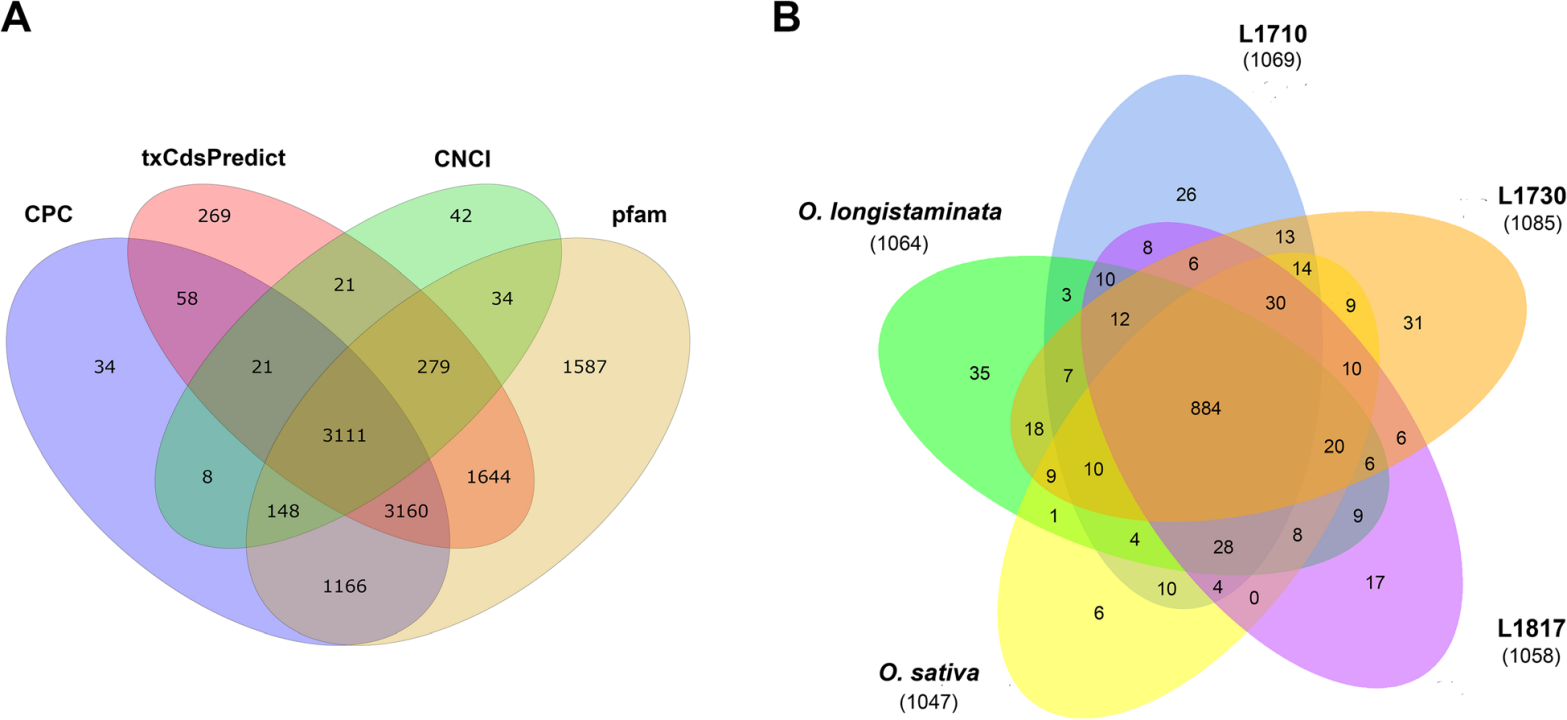
Venn diagram of lncRNAs predicting by four methods (**a**) and Venn diagram of the number of lncRNAs expressed in five lines (**b**). Three software CPC, txCdsPredict, CNCI and a protein database pfam were used to predict lncRNAs, and the transcript was determined when at least three of the four methods were consistent

**Fig. 2:**
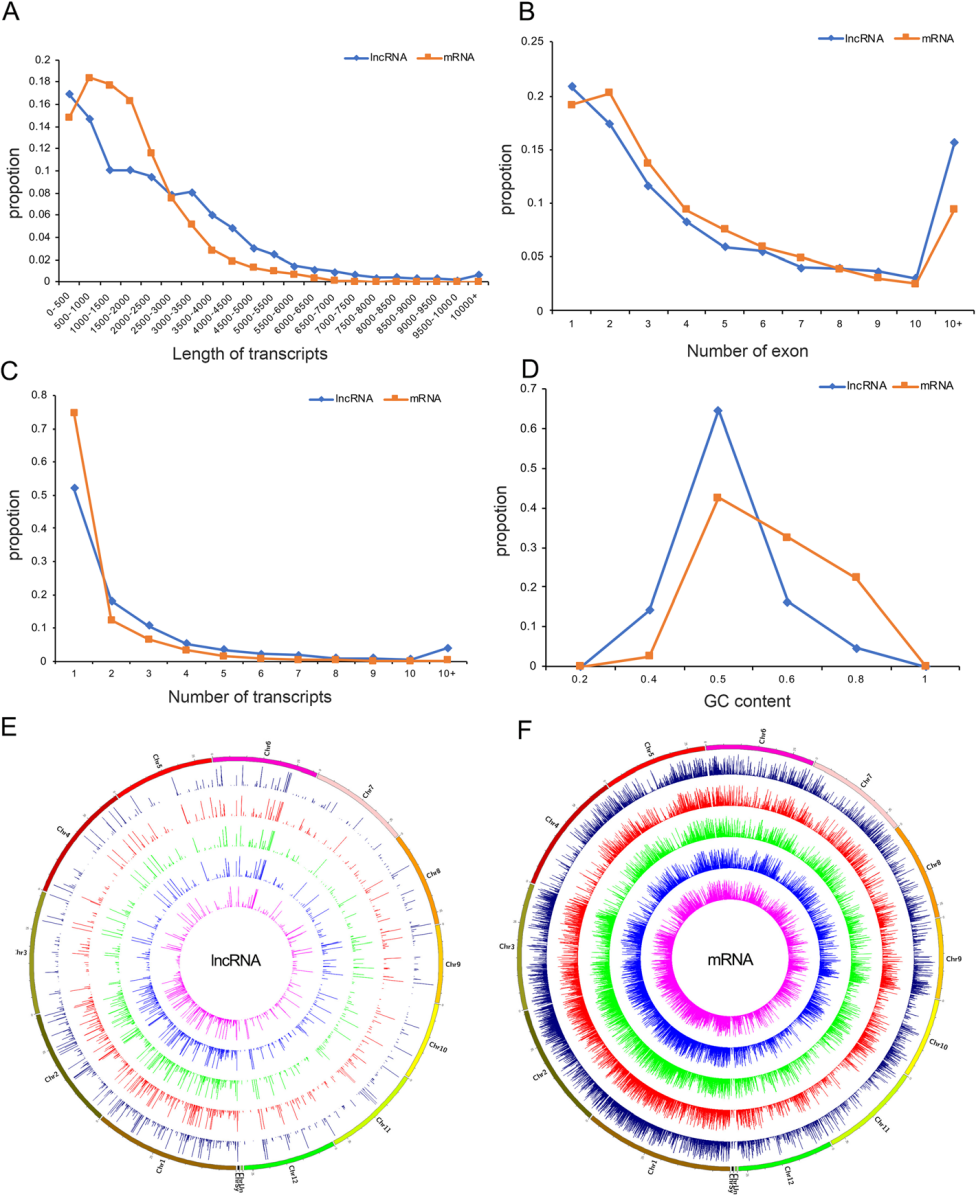
The comparative characteristics analysis of expressed lncRNAs and mRNAs. The length distribution (**a**) and exon number (**b**), the transcript number (**c**), GC content (**d**) of lncRNAs and mRNAs encoding genes. The distribution of expressed lncRNAs (**e**) and mRNAs (**f**) in twelve chromosomes

To identify lncRNAs in *O. sativa*, *O. longistaminata* and their three BIL progenies, the coding ability of the 16,038 novel transcripts were predicted using three software (CPC, CNCI and txCdsPredict) and Pfam protein database. A total of 6719 novel lncRNAs were identified by predicting their coding ability (Fig. 1a), and then 1254 novel lncRNAs were screened out by the quantitative analysis with RSEM software (Fig. 1b, Table 1 and Supplementary Table S3). In addition, the sequence characteristics of identified lncRNAs were performed through comparing with that of mRNAs. The length of lncRNAs varied from 200 to 18,313 bp with an average of 2348 bp, which was longer than that of known mRNAs (an average of 1708 bp) (Fig. 2a). About 50% of lncRNAs were more than 2000 bp in length, of which 42 lncRNAs were longer than 10,000 bp. The number of exons of the genes encoding lncRNAs was basically consistent with that of the known mRNA-coding genes, and 49.8% of lncRNA-coding genes and 53.2% of the known mRNA-coding genes contained 1–3 exons respectively (Fig. 2b). Most known mRNAs (87.1%) and lncRNAs (70.1%) were derived from genes having one or two transcripts (Fig. 2c). The GC content of lncRNA-coding genes varied from 23.45 to 78.93% with an average of 46%, and most of them (79%) with the GC content less than 50% (Fig. 2d), while the GC content of known mRNA-coding genes varied from 28.73 to 84.16% with an average of 52.88, and 55% of them with the GC content more than 50%. Overall, the above results showed that the characteristics of lncRNA and mRNA sequences were diversified, for the length of lncRNAs was longer than that of known mRNAs, but the exon number of genes encoding lncRNAs was less than that of genes encoding mRNAs, and the GC content of lncRNA-encoding genes was also lower than that of known mRNA-encoding genes.

**Table 1:** The number of lncRNAs and mRNAs expressed *O. sativa*, *O. longistaminata* and their three BIL progenies

Sample	Number of lncRNAs	Number of mRNAs
L1710	1069	23,023
L1817	1058	23,072
L1730	1085	22,709
*O. sativa*	1047	22,448
*O. longistaminata*	1064	23,781

The expression level of lncRNAs and mRNAs were calculated using RSEM software. The number of lncRNAs and mRNAs expressed in *O. sativa*, *O. longistaminata* and their three BIL progenies was shown in Table 1, and the specific FPKM values for them were shown in Supplementary Table S3. As shown in Fig. 1b, 70.5% (884 of 1254) of lncRNAs expressed in all five lines. The number of lncRNAs which only expressed in one line was the most (35) in *O. longistaminata* and the least (6) in *O. sativa* (Fig. 1b). Furthermore, the distribution of expressed lncRNAs and mRNAs on 12 chromosomes was visualized using Circos (Fig. 2e & f). The results showed that over 50% lncRNAs and over 40% mRNAs were expressed on chromosomes 1 and 2, and the percentage of expressed lncRNAs was higher than that of expressed mRNAs on chromosomes 1, 2 and 11.

### Differentially expressed lncRNAs in five lines

**Fig. 3:**
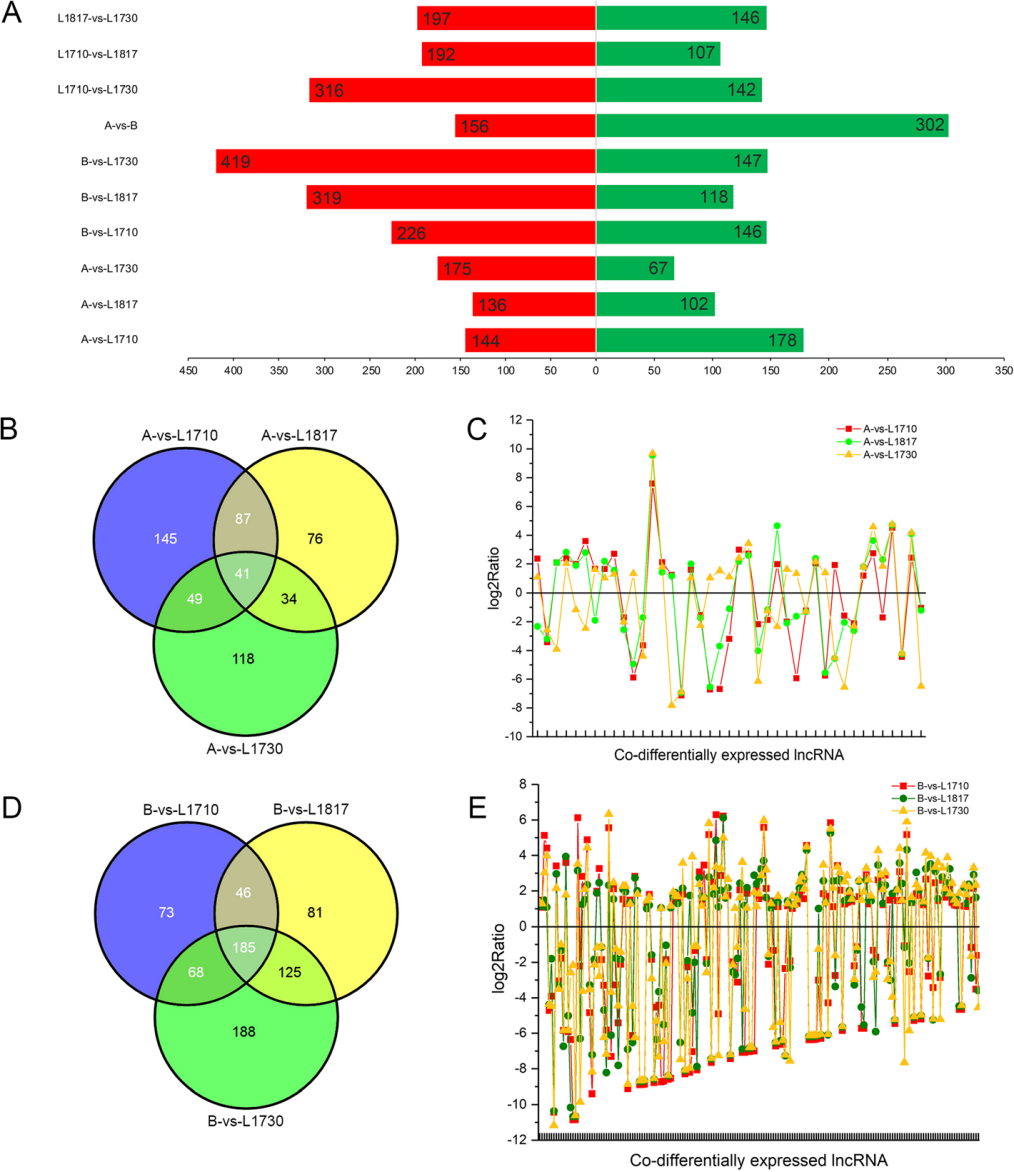
Analysis of DE-lncRNAs. Number of DE-lncRNAs in all comparison groups (**a)**. The red and green bars represented up- and down-expressed lncRNAs, respectively. Venn diagram of common DE-lncRNAs among three progenies and *O. sativa* (**b)** and the FC distribution of them (**c**). Venn diagram of common DE-lncRNAs among three progenies and *O. longistaminata* (**d)** and the FC distribution of them (**e**)

The FPKM values of lncRNAs and mRNAs (Supplementary Table S3) were used to analyze the differential expression of lncRNAs/mRNAs among *O. sativa*, *O. longistaminata* and their three BIL progenies with |log_2_FC| ≥ 1 and FDR ≤ 0.001 by DEseq software. The analysis of the differentially expressed lncRNAs (DE-lncRNAs) was shown in Fig. 3. DE-lncRNAs were mostly up-regulated in progenies compared with their parents (Fig. 3a). Furthermore, the DE-lncRNAs between progenies and *O. longistaminata* (an average of 458, and 69.2% of them were up-regulated in progenies) were significantly higher than that of progenies vs. *O. sativa* (an average of 267, and 58.1% of them were up-regulated in progenies). Meanwhile, 41 and 185 common DE-lncRNAs were discovered in all three progenies compared with their two parents, respectively (Fig. 3b & d). Among these identified common DE-lncRNAs, the number of up-regulated lncRNAs were also higher than down-regulated lncRNAs in progenies (Fig. 3c & e). The above results indicated that there was a greater difference between the three BIL progenies and the parent *O. longistaminata*, and the up-regulated DE-lncRNAs in BIL progenies might play critical roles. In the difference analysis among the three progenies, 299 DE-lncRNAs were found in L1710 vs. L1817, and 458 were found in L1710 vs. L1730 (Fig. 3a). This phenomenon was consistent with the fact that L1710 and L1730 have the largest difference in plant height. For a more detailed analysis, DE-lncRNAs with different fold changes (FC > 2, FC > 10, FC > 50, FC > 100, FC > 200) in the three progenies compared with their parents were counted (Supplementary Fig. S2). With the increase of the FC of DE-lncRNAs, more lncRNAs were found in the comparison group with higher difference in plant height between progeny and parents. For example, among the comparison groups of three progenies and *O. sativa*, the number of DE-lncRNAs with FC > 2 was the most in L1710, while the number of DE-lncRNAs with FC > 50/100/200 was the largest in L1730.

### Prediction of target protein-coding genes of lncRNAs and their GO analysis

**Fig. 4:**
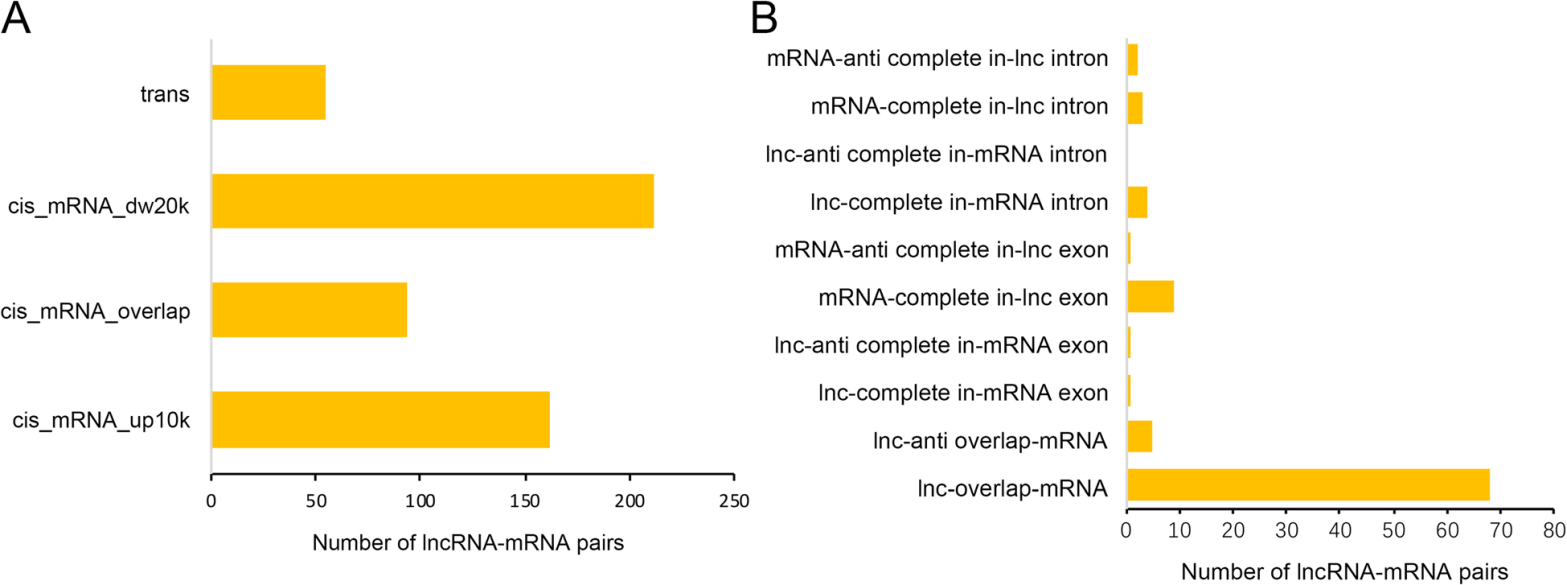
The regulatory relationship types of lncRNA-mRNA pairs. Histograms of 4 classes of lncRNAs regulating target protein-coding genes (**a**) and 10 subclasses of Class cis_mRNA_overlap (**b**). Subclass lnc-anti complete in-mRNA intron was not detected in this study

One way in which lncRNAs perform their biological function is to regulate the expression of protein-coding genes through *cis* or *trans* interactions. LncRNAs may regulate the expression of target genes by one *trans*-regulated and three *cis*-regulated ways (cis_mRNA_up10k, cis_mRNA_overlap and cis_mRNA_dw20k) (Fig. 4a). The Class cis_mRNA_overlap could be further divided into 10 subclasses (Fig. 4b). Totally, the present data showed 89.5% (468 of 523) of lncRNAs regulated the expression of target genes through *cis*-acting, among which 45.3% (212 of 468) belonged to the cis_mRNA_dw20k regulatory class. These results suggested that *cis*-regulation rather than *trans*-regulation was the main regulation type, and *cis*-regulation of lncRNAs located at 20 kb downstream of the target genes was the common *cis*-regulation type among the predicted lncRNA-mRNA regulation pairs in this study. As shown in Supplementary Table S4, a total of 431 lncRNA-mRNA regulation pairs were detected in *O. sativa*, *O. longistaminata* and their three BIL progenies, among which 373 mRNAs were potential targets of 297 lncRNAs, indicating that some lncRNAs may regulate multiple mRNAs at the same time.

**Fig. 5:**
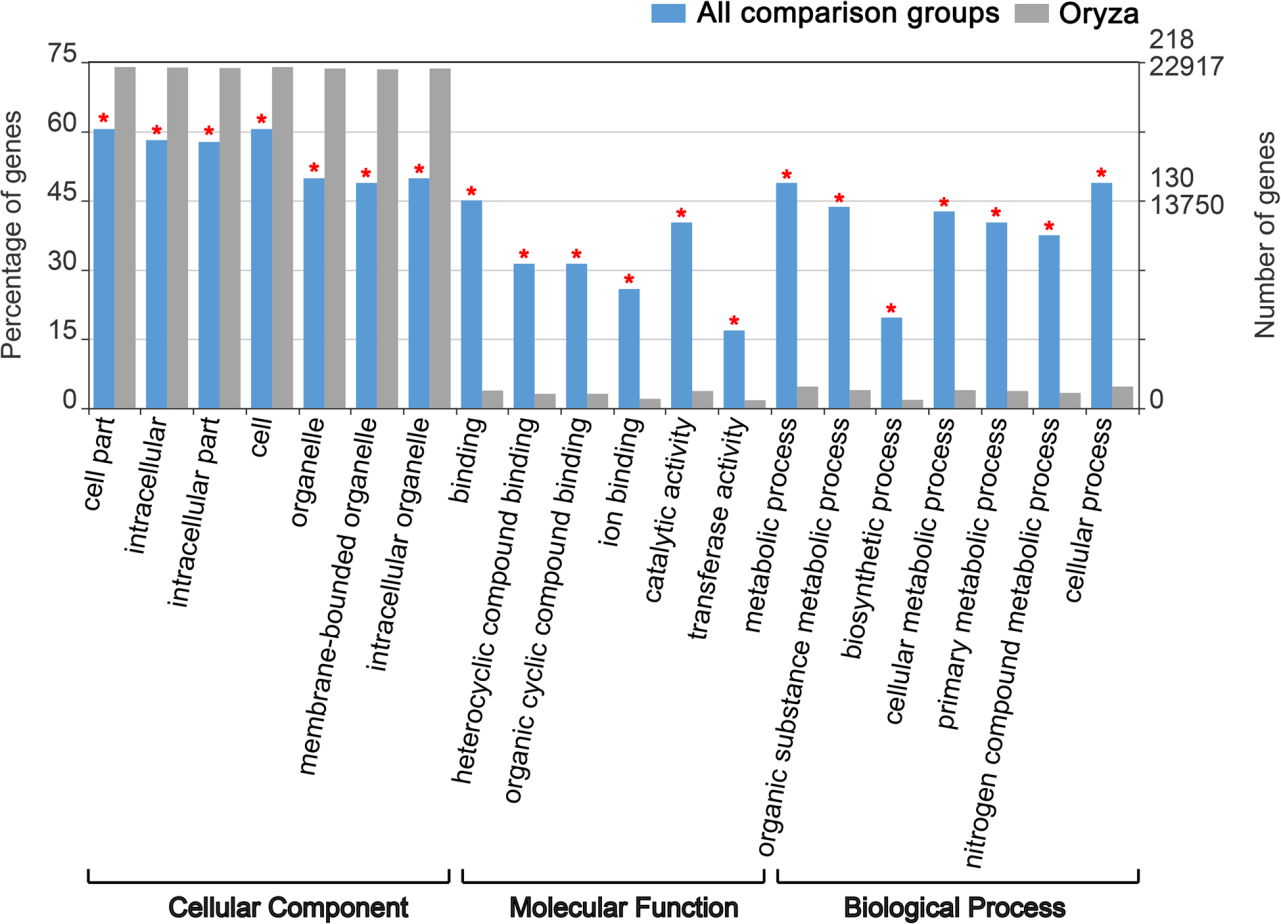
The GO classifications of predicted target genes of DE-lncRNAs in all comparison groups. Red mark ‘*’ indicated significantly enriched GO terms, of which the *P*-value was below 0.001

In order to further understand the role of DE-lncRNAs among *O. sativa*, *O. longistaminata* and their three BIL progenies, GO enrichment analysis was conducted on the target genes of DE-lncRNAs in all comparison groups with rice genome as the reference (Fig. 5). Results showed that potential target genes of DE-lncRNAs were significantly enriched (*P* < 0.001) in 7, 6 and 7 GO terms of cellular component, molecular function and biological process categories, respectively (Fig. 5). In addition, the percentage of potential target genes of DE-lncRNAs was lower than that of rice genome (the background) in significantly enriched GO terms of cellular component category, while higher in significantly enriched GO terms of molecular function and biological processes categories. This phenomenon indicated that DE-lncRNAs target genes that are significantly enriched in molecular function and biological processes categories might play important roles in regulating the growth and development of *O. sativa*, *O. longistaminata* and their three BIL progenies.

### Analysis of lncRNA acting as precursors of miRNAs

**Fig. 6:**
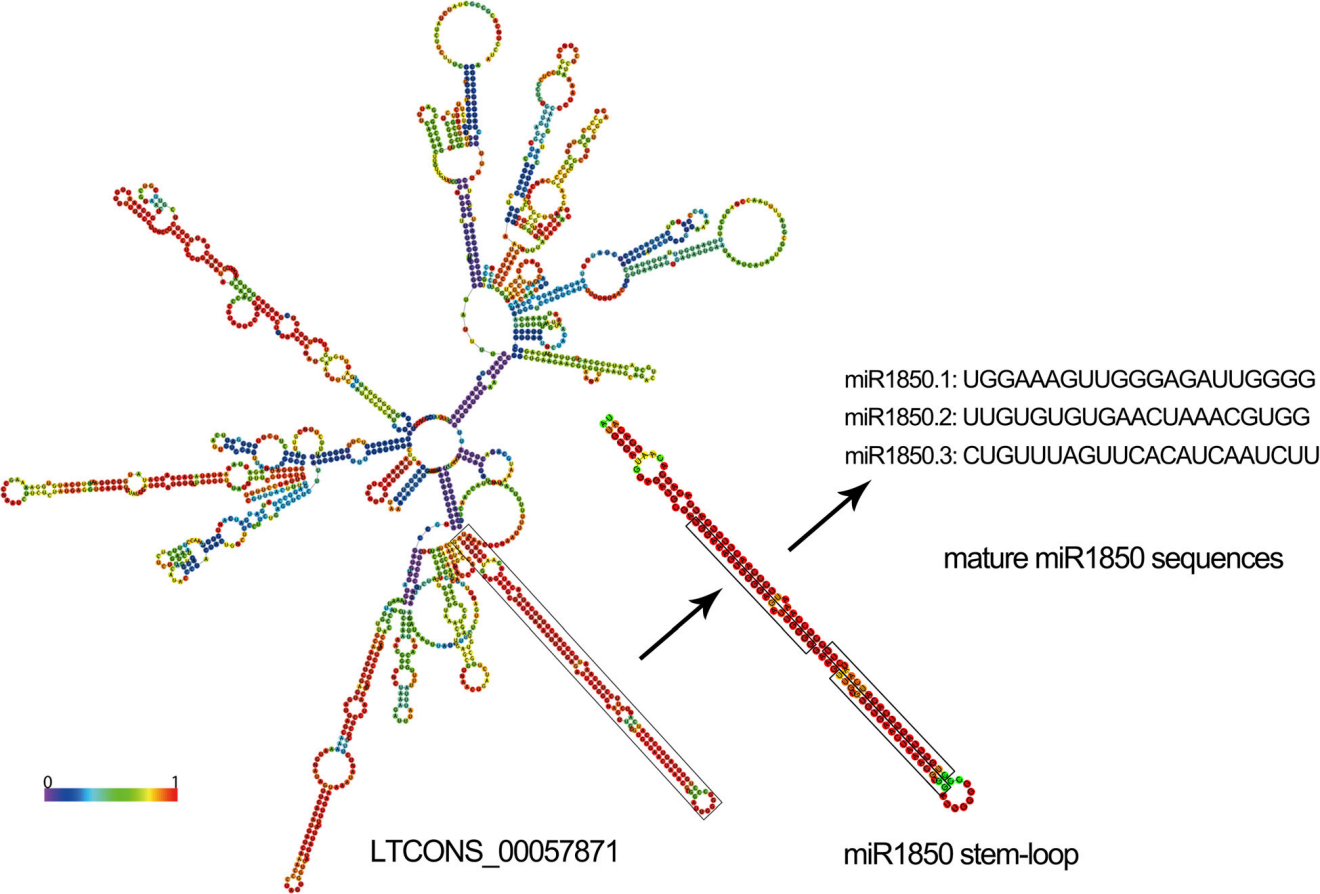
The predicted secondary structure of lncRNA as the putative miRNA precursor and miRNA (take LTCONS_00057871 and miR1850 for instance)

LncRNAs could act as the precursors of microRNAs (miRNAs). To screen for miRNAs precursors in the five lines, the sequences of lncRNAs were aligned to the miRbase database using BLAST. As shown in Table 2, a total of 22 expressed lncRNAs were predicted to be the precursor of 20 miRNAs, of which 18 lncRNAs were DE-lncRNAs in five lines. Most lncRNAs acted as the precursor of one miRNA, while two of which (LTCONS_00035053, LTCONS_00007959) served as precursors of more than one miRNA (Table 2). Moreover, several lncRNAs might also be precursors for the same miRNA. For example, both LTCONS_00034708 and LTCONS_00034707 were the precursor of miR396c (Table 2). In addition, more than 50% of lncRNAs predicted as the precursors of miRNA were found to be transcribed from chromosome 2. RNAfold web server was used to predict the secondary structures of lncRNAs and miRNA precursors to visualize the relationship of them. The predicted secondary structure of LTCONS_00057871 contained multiple stem-loop structures, and one of which was the potential precursor of miR1850 (Fig. 6). Mature miRNAs (miR1850.1, miR1850.2 and miR1850.3) were finally formed after the precursor was processed by enzymes.

**Table 2:** Prediction of lncRNA as miRNA precursor

miRNA ID	lncRNA ID	Alignment length (nt)	Alignment ratio	Location
miR162a	LTCONS_00029262^a^	171	1	Chr2
miR166f	LTCONS_00014145^a^	107	0.972	Chr10
miR169a	LTCONS_00001063^a^	173	1	Chr1
miR394	LTCONS_00029635^a^	110	1	Chr2
miR396c	LTCONS_00034708^a^	141	1	Chr2
miR396c	LTCONS_00034707^a^	141	1	Chr2
miR319b	LTCONS_00000824	197	1	Chr1
miR166k	LTCONS_00035053	127	1	Chr2
miR166h	LTCONS_00035053	119	1	Chr2
miR393b	LTCONS_00051533^a^	132	0.977	Chr4
miR172d	LTCONS_00034806^a^	130	1	Chr2
miR172d	LTCONS_00034805^a^	130	1	Chr2
miR1430	LTCONS_00024512^a^	139	0.958	Chr12
miR444b	LTCONS_00033178^a^	138	1	Chr2
miR444b	LTCONS_00033177	138	1	Chr2
miR444d	LTCONS_00034231^a^	143	1	Chr2
miR1848	LTCONS_00027106^a^	69	0.921	Chr2
miR1850	LTCONS_00057871^a^	133	0.985	Chr5
miR1428b	LTCONS_00007959^a^	124	1	Chr1
miR1428d	LTCONS_00007959^a^	124	0.919	Chr1
miR1846e	LTCONS_00081175^a^	69	0.957	Chr9
miR1846e	LTCONS_00057746	69	0.928	Chr5
miR396f	LTCONS_00034891^a^	176	1	Chr2
miR5083	LTCONS_00009921^a^	380	1	Chr1

^a^indicated significantly differentially expressed lncRNAs

### Analysis of lncRNA acting as decoys or target mimics of miRNAs

**Fig. 7:**
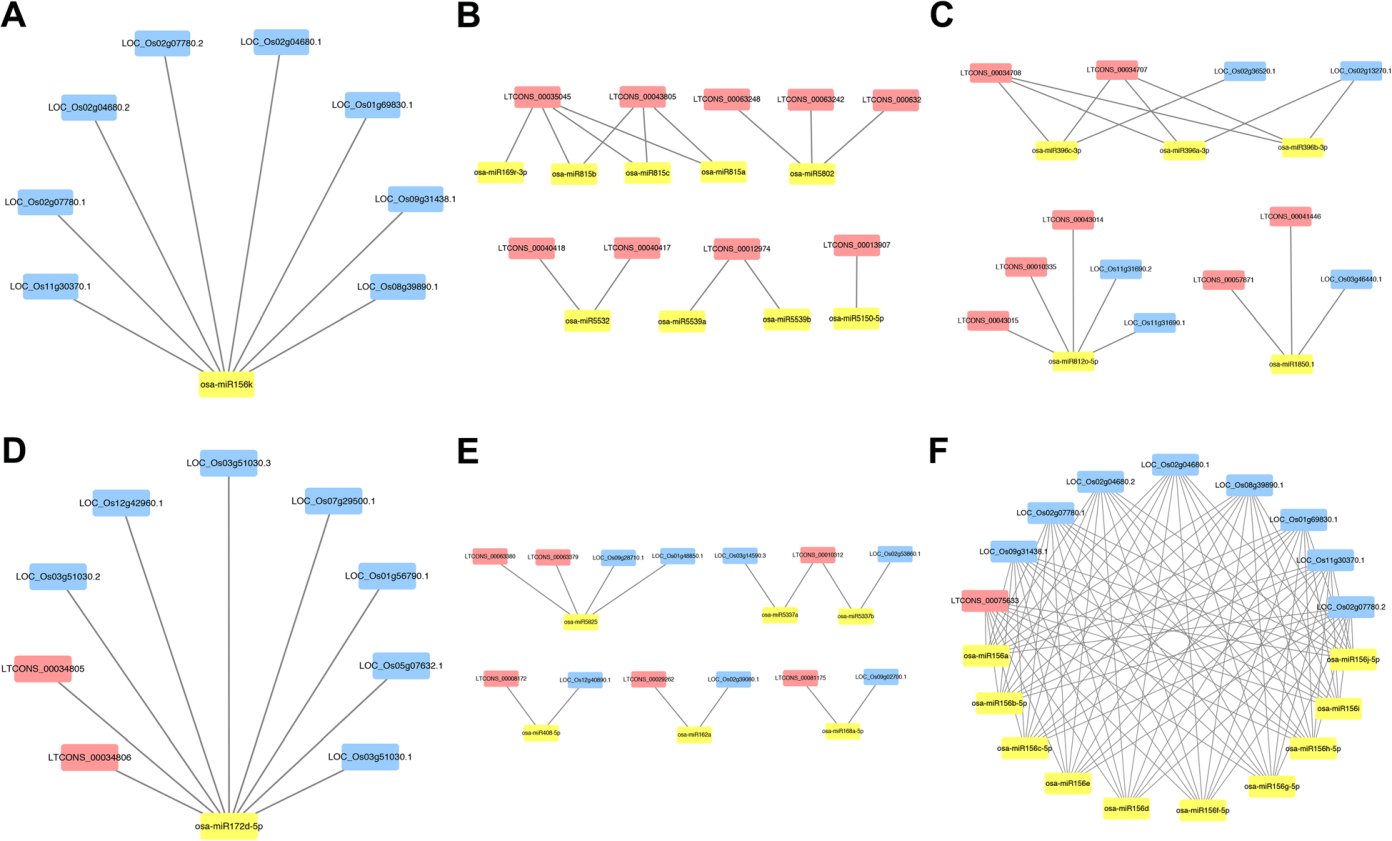
The typical networks of lncRNAs, miRNAs and mRNAs

LncRNAs could be used as decoys to directly or indirectly regulate the expression of target genes [30]. To further explore the roles of three kinds of RNAs (lncRNA, miRNA, and mRNA) in *O. sativa*, *O. longistaminata* and their three BIL progenies, interaction networks were constructed based on previous miRNA sequencing studies [2]. A total of 78 lncRNAs and 271 mRNAs were targeted by 280 miRNAs in the network (Supplementary Table S5). Statistic showed that 72.2% (202 of 280) miRNAs only targeted mRNAs (Supplementary Table S5). For example, osa-miR156k targeted 8 mRNAs (Fig. 7a), and three of them were found to be important in rice growth. Specifically, two mRNAs (LOC_Os02g04680.1 and LOC_Os02g04680.2) were the two transcripts of *OsSPL3 (SQUAMOSA PROMOTER-BINDING PROTEIN-LIKE3)*, which regulated root crown development in rice [31]. The other mRNA (LOC_Os08g39890.1) were the transcript of *OsSPL14/IPA1*, which could regulate *DEP1* (*DENSE AND ERECT PANICLE1*), a critical gene influencing the plant height and panicle length [32]. Moreover, only a small fraction of miRNAs (3.2%, 9 of 280) only targeted lncRNAs (Supplementary Table S5, Fig. 7b). There were 24.7% (69 of 280) miRNAs targeted both mRNAs and lncRNAs (Supplementary Table S5). Further analysis found that only 5 miRNAs targeted more lncRNAs rather than mRNAs (Fig. 7c) and most miRNAs targeted more mRNAs rather than lncRNAs (84.1%, 58 of 69) when miRNAs target both mRNAs and lncRNAs, which indicated that the binding ability of lncRNAs to miRNAs was weaker than that of mRNAs in most cases. For example, osa-miR172d-5p targeted 2 lncRNAs and 7 mRNAs (Fig. 7d), among which 3 mRNAs (LOC_Os03g51030.1, LOC_Os03g51030.2 and LOC_Os03g51030.3) were the three transcripts of *PHYA* (*Phytochrome A*), which played multiple roles in controlling the internode elongation in rice [33]. In addition, there were 6 miRNAs targeted with equal amounts of mRNAs and lncRNAs (Fig. 7e). Different miRNAs might also regulate multiple identical targets simultaneously (Fig. 7f). Interestingly, seven miRNAs were found to target their own potential precursor lncRNAs (Table 3), suggesting that these lncRNAs not only act as precursors of miRNAs, but also can bind with them to participate in the regulation of target gene expression.

**Table 3:** Analysis of miRNA targeting its precursor lncRNA

miRNA ID	Targets
miR162a	LTCONS_00029262^a^
miR393b	LTCONS_00051533^a^
miR396c	LTCONS_00034708^a^
	LTCONS_00034707^a^
miR172d	LTCONS_00034806^a^
	LTCONS_00034805^a^
miR1850	LTCONS_00057871^a^
	LTCONS_00041446
miR444b	LTCONS_00033178^a^
	LTCONS_00033177^a^
	LTCONS_00034231
miR444d	LTCONS_00034231^a^
	LTCONS_00033177
	LTCONS_00033178

^a^indicated lncRNA acts as both precursor and target of corresponding miRNA

### Parental expression level dominance analysis in three progeny lines

**Fig. 8:**
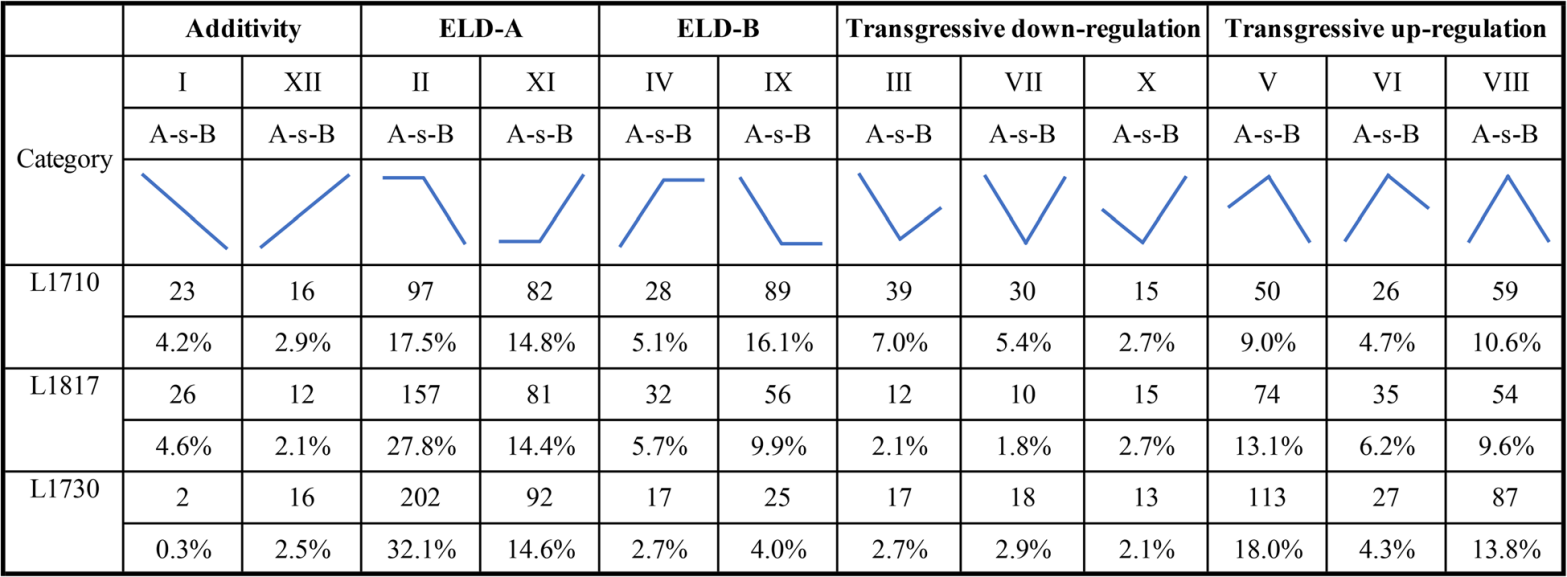
Twelve expression patterns of lncRNAs in three progenies. A and B stand for *O. sativa* and *O. longistaminata*, respectively

Expression level dominance (ELD) refers to the expression level of some genes in the progeny close to that of one parent, but different from that of the other parent. Recently, many studies about hybrids and their parents have found that the expression of mRNAs showed parental ELD in progenies [2, 29, 34]. According to the criteria defined by Yoo et al. [34], gene expression patterns are divided into 12 categories, as shown in Fig. 8. Over 50% of lncRNAs showed parental ELD (category II, XI, IV and IX), and about 40% of lncRNAs showed transgressive up/down-regulation (category III, VII, X, V, VI and VIII) in all three progenies. Moreover, the number of lncRNAs showed parental ELD-A (A stands for *O. sativa*) was higher than that showed parental ELD-B (B stands for *O. longistaminata*) in all three progenies. To further get a glimpse of the possible biological functions of lncRNAs with ELD expression patterns, their potential target mRNAs were used for GO enrichment analysis. As shown in Supplementary Table S6, about 30% of lncRNAs had potential target mRNAs in each progeny. The number of targets of lncRNAs showed ELD-A was higher than that showed ELD-B in all three GO categories (cellular component, molecular function and biological process) of L1817 and L1730 (Supplementary Fig. S3). However, the number of targets of lncRNAs showed ELD-A was less than that showed ELD-B in all three GO categories of L1710. In addition, ELD-A lncRNAs targets were enriched in ‘cell’, ‘cell part’ and ‘organelle’ term, while ELD-B lncRNAs targets were enriched in ‘membrane’ term in all three progenies (Supplementary Fig. S3). Furthermore, ELD-A lncRNAs targets were enriched in ‘binding’ term in L1710, while ELD-B lncRNAs targets were enriched in this term in L1817 and L1730 (Supplementary Fig. S3). ELD-A lncRNAs targets were enriched in ‘cellular process’ term in L1710 and L1730, while ELD-B lncRNAs targets were enriched in this term in L1817 (Supplementary Fig. S3). ELD-A lncRNAs targets were enriched in ‘metabolic process’ term in L1730, while ELD-B lncRNAs targets were enriched in this term in L1710 and L1817 (Supplementary Fig. S3). In conclusion, the non-addictive genes accounted for the majority in the comparison between BIL progenies and their parents, moreover, all three progenies (L1710, L1817 and L1730) biased towards *O. sativa*.

### Validation of the data by qRT-PCR

**Fig. 9:**
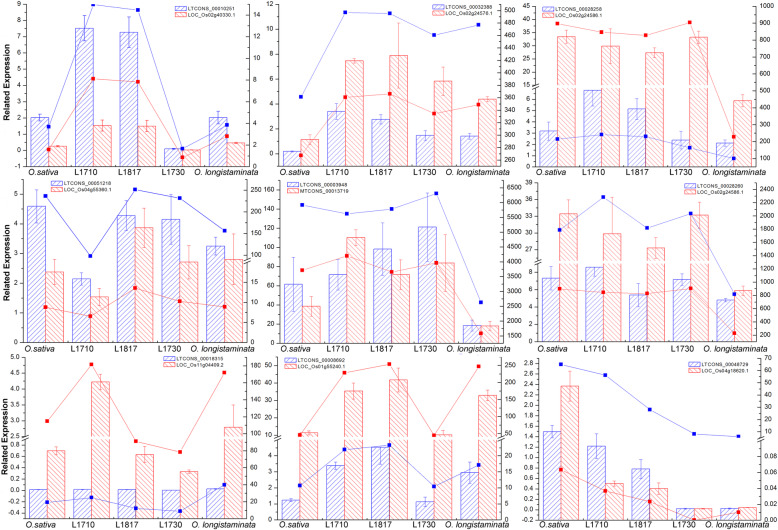
Nine lncRNAs and their target genes were selected to verify the accuracy of the sequencing data using qRT-PCR in *O. sativa*, *O. longistaminata* and their three BIL progenies. The bar graph showed the results of qRT-PCR, and the broken line graph showed the results of sequencing, with lncRNAs in blue and mRNAs in red

To verify the accuracy of the sequencing data, 9 lncRNAs and their potential target genes were selected randomly for qRT-PCR analysis. Results showed that the sequencing data and the qRT-PCR results were basically consistent (Fig. 9), indicating that the sequencing data were reliable. The primers used for qRT-PCR were listed in Supplementary Table S7.

## Discussion

In recent years, lncRNAs, a kind of regulatory RNAs, have become a research hotspot. With the development of sequencing technology, lncRNAs have been found to play critical roles in plant growth and sexual reproduction [14, 35]. Specifically, studies have shown that lncRNAs were involved in female/male sterility of plants [36–39], and plant stress response process [40, 41]. So far, there have been no reports on the lncRNA expression patterns and their functions in BIL progenies relative to their parents. Based on our previous studies on the mRNA and miRNA expression patterns in *O. sativa*, *O. longistaminata* and their three BIL progenies [2], the study on lncRNA expression patterns will help us to further understand the regulatory factors for differential expression of genes in BIL progenies relative to their parents.

### LncRNAs might be involved in regulating the expression of plant hormone-related genes in *O. sativa*, *O. longistaminata* and their BIL progenies

Plant hormones can regulate the physiological responses of plant growth, development and differentiation independently or in a variety of coordinated ways. Plant hormone content are affected by related gene expressions, while the later are regulated at transcriptional and post-transcriptional levels by various factors [29, 42, 43]. Many lncRNAs with down-regulated polyadenylation (DPA) participated in the biosynthesis, transport and metabolism of ABA in rice, thereby activating the expression of a series of stress response genes [44]. As described below, some lncRNAs targeted gibberellin (GA), ethylene and auxin related genes in this study, and their regulatory effects might have an impact on growth and development of rice. LTCONS_00063919 targeted *D35/OsKO2* (LOC_Os06g37364, Supplementary Table S4), which encodes an *ent*-kaurene oxidase (KO) of catalytic gibberellin biosynthesis, and rice without this gene shows a severe dwarf-phenotype [45]. *SLRL1* (LOC_Os01g45860), a member of *GRAS* gene family, was targeted by LTCONS_00002962 (Supplementary Table S4), and GA induces the expression of *SLRL1*, the overexpression of which will also lead to the dwarf-phenotype in rice [46]. *GID2* (LOC_Os02g36974) was a target gene of LTCONS_00033182 (Supplementary Table S4), which regulate the degradation of an inhibitory factor (SLR1) in GA signal transduction, and rice presented a severe dwarfing phenotype when *GID2* gene was mutated [47]. Furthermore, LTCONS_00032876 was predicted to target *OsCTR2* (LOC_Os02g32610, Supplementary Table S4), and the Raf-like protein CONSTITUTIVE TRIPLE RESPONSE1 (CTR1) was involved in ethylene receptor signal transduction to regulate multiple growth and development processes in rice [48]. In addition, *OsARF24* was a target gene of LTCONS_00025454 (Supplementary Table S4), and the low expression of *OsARF23* and *OsARF24* would reduce the response of rice to auxin, thereby affecting the growth and morphogenesis of rice [49]. Therefore, lncRNAs that target to plant hormone-related genes might regulate plant growth and development and affect the plant height in *O. sativa*, *O. longistaminata* and their BIL progenies.

### LncRNAs might regulate the adaptability of *O. sativa*, *O. longistaminata* and their BIL progenies

Studies have shown that lncRNAs are involved in plant responses to various biotic and abiotic stresses [40, 44, 50]. Jain et al. [41] identified many lncRNA candidate genes from 24 blast resistant rice lines, revealing their regulatory roles in rice blast resistance. Many lncRNAs with DPA may play important roles in the growth of rice under various abiotic stresses (such as heat, cold, drought and salt stress) [44]. As shown in Supplementary Table S4, LTCONS_00033755 targeted *trehalose-6-phosphate phosphatase* (*OsTPP1*, LOC_Os02g44230), which was a member of *TPS/TPP* gene family. The over-expression of *OsTPP1* could enhance the tolerance to cold and salt stress, and simultaneously activated the expression of multiple stress response genes in rice [51]. The protein encoded by *GF14e* (LOC_Os02g36974, a targeted gene of LTCONS_00033182, Supplementary Table S4) affects the expression of defense response genes, cell death and resistance to bacterial blight and sheath blight in rice [52]. Meanwhile, the protein encoded by *OsGF14e*, which was regulated by WRKY71, positively regulated rice resistance to panicle blast [53]. In addition, *WRKY13* (LOC_Os01g54600) was targeted by LTCONS_00010291 (Supplementary Table S4) in this study, and it is well known that WRKY13 could directly inhibited WRKY42, which could negatively regulate the response of rice to blast fungus (*Magnaporthe oryzae*) by inhibiting JA signaling related genes [54]. Moreover, *OsPCF5* was targeted by LTCONS_00010204 (Supplementary Table S4), and as a member of TCP transcription factor family, OsPCF5 plays a negative role in response to low temperature stress in rice [55]. Thus, four lncRNAs mentioned above might regulate the expression of disease-resistance related genes in *O. sativa*, *O. longistaminata* and their BIL progenies, and thereby affecting their adaptability.

### LncRNAs might regulate the growth of *O. sativa*, *O. longistaminata* and their BIL progenies by competitively binding miRNAs to mRNAs

Some lncRNAs bind to miRNAs as decoys, so that these miRNAs cannot bind to their target genes normally, thereby affecting their regulation of target genes [20]. Two lncRNAs were found to adsorb miR160 and miR164 respectively in anther, pistil and seed of rice [35]. In this study, LTCONS_00001063 was predicted to competitively bind miR169f.1 and miR169o with *OsHAP2G* (LOC_Os07g41720) and *OsHAP2H* (LOC_Os03g44540), which were the two coding genes of the HAP2 subunit of HAP complex [56]. Thus, it is speculated that LTCONS_00001063 might participate in the regulation of the HAP2 subunit gene expression during the growth and development of rice. Moreover, LTCONS_00034806 and LTCONS_00034805 were predicted to competitively bind miR172d-5p with *Phytochrome A* (*PHYA*; LOC_Os03g51030), which can regulate the elongation of rice nodes and play critical role in the vegetative growth stage of rice [33, 57]. Therefore, LTCONS_00001063, LTCONS_00034806 and LTCONS_00034805 may regulate the expression level of miRNA target genes by competitively binding miRNAs with specific mRNAs, and then participate in the growth process of stem at the jointing stage of rice.

### The difference of lncRNA expression between BIL progenies and *O. sativa* is smaller than that between BIL progenies and *O. longistaminata*

Our previous study mainly explored the gene expression and miRNA regulation in *O. sativa*, *O. longistaminata* and their three BIL progenies [2]. In this study, expression characteristics of lncRNAs and their potential target genes were investigated in these species. These two studies found something in common. For example, the previous study showed that the number of DEGs between progenies and *O. longistaminata* was higher than that between progenies and *O. sativa*. Similarly, the number of DE-lncRNAs between progenies and *O. longistaminata* was also higher than that between progenies and *O. sativa* in this study. In addition, the previous study showed that most genes displayed ELD of one parent and more ELD-A (A stands for *O. sativa*) genes than ELD-B (B stands for *O. longistaminata*) genes were observed in three progenies. Analogously, over 50% of lncRNAs showed parental ELD and the number of lncRNAs showed parental ELD-A was higher than that showed parental ELD-B in all three progenies in this study. The above results indicated that the difference of lncRNA expression between BIL progenies and *O. sativa* is smaller than that between BIL progenies and *O. longistaminata*. As indicated in previous studies, most of the chromosome complements of the BIL progenies were inherited from the recurrent parent *O. sativa* [2]. Therefore, the most likely reason for the difference of lncRNA expression was the genetic background difference between BIL progenies and each of their parent.

## Conclusions

In this study, high-throughput ss-RNA-seq technology was used to explore the functional characteristics and difference of lncRNAs in *O. sativa*, *O. longistaminata* and their three BIL progenies. LncRNAs were identified and most of them expressed in all five lines. The analysis of DE-lncRNAs showed that the difference of lncRNAs between the three progenies and *O. sativa*, including the number and the fold change of DE-lncRNAs, was greater than that between the three progenies and *O. longistaminata*. In addition, most DE-lncRNAs were up-regulated in progenies compared with their parents. Some lncRNAs could regulated the expression of mRNAs, most of which was *cis*-regulation. Most miRNAs targeted more mRNAs rather than lncRNAs when both mRNAs and lncRNAs were targeted. Some miRNAs were found to target their own potential precursor lncRNAs. Furthermore, more than half of the lncRNAs showed parental ELD in all three progenies, and the number of lncRNAs showed parental ELD*-O. sativa* was higher than that showed parental ELD-*O. longistaminata*, which indicated that all three progenies were biased towards *O. sativa*. Further analysis showed that lncRNAs might be involved in regulating the expression of plant hormone-related genes in all five lines, and also can regulate the adaptability of them. Taken together, these results provided valuable clues for elucidating the functional features and expression differences of lncRNAs between *O. sativa*, *O. longistaminata* and their BIL progenies, and expanded our understanding of the biological functions of lncRNAs in rice.

## Methods

### Plant materials

*Oryza sativa ssp. indica* cv. 9311, *O. longistaminata* and their three BILs (BC_2_F_12_) progenies (L1710, L1817 and L1730) were used as plant materials in this study. These materials were all obtained from Dr. Shaoqing Li’s laboratory in College of Life Sciences, Wuhan University, Wuhan, China. The construction of BC_2_F_12_ consists of 4 steps: 1) *O. sativa* (maternal) was crossbred with *O. longistaminata* (paternal) to generate F1 hybrid; 2) F1 hybrid (paternal) was backcrossed with *O. sativa* (maternal) to generate BC_1_F_1_; 3) BC_1_F_1_ individuals (paternal) were backcrossed with *O. sativa* (maternal) to generate BC_2_F_1_; 4) BC_2_F_1_ finally produces BC_2_F_12_ through 11 generations of self-fertilization by the single seed descent method. The genome composition of three progenies was basically the same, and most of which were inherited from the cultivated rice (*O. sativa*), while only about 10 to 15% of which was inherited from *O. longistaminata* [2]. As Cao et al. [2] described, three BIL progenies had different plant heights. Specifically, the plant height of 5 lines at mature stage was ranked as L1710 < *O. sativa* < L1817 < *O. longistaminata* < L1730 [2]. Germinated seeds of progenies and *O. sativa* were sown in soil and then the seedlings were transplanted into plots after 30 days in the greenhouse of Wuhan University, Wuhan, China. The rhizomes of *O. longistaminata* were also planted in plots. Stems from five lines with three biological replicates at jointing-booting stage were harvested and immediately stored in liquid nitrogen for subsequent RNA extraction.

### Construction of RNA libraries and sequencing

The total RNAs of stems in five lines were extracted using TRIzol reagent according to the manufacturer’s protocal (Invitrogen, Carlsbad, CA, USA). The purity, concentration and OD_260_/OD_280_ ratio of total RNAs in each sample were detected by Agilent 2100 Bioanalyzer (Agilent RNA 6000 Nano Kit). The ribosomal RNAs (rRNAs) were removed from the total RNAs using the Ribo-Zero™ rRNA removal kit, and then the RNAs were randomly fragmented. The first-strand cDNA was synthesized by reverse transcription with the fragmented RNA as the template and a random six-base sequence as the primer, and the second-strand cDNA was subsequently synthesized by replacing dTTP with dUTP. The sequencing libraries were then constructed through the cDNA end repair, adding poly A-tailing and adapter, Uracil-N-Glycosylase digestion, and several rounds of PCR amplification. Quality control and quantification analysis were performed on all libraries. Finally, the fifteen libraries were sequenced using the Illumina HiSeq 4000 platform and 150 bp paired-end reads were generated. Each line was sequenced with three biological replicates.

### Data filtering, sequence alignment and assembly

To further ensure that rRNA was not presented in raw reads, reads were aligned to the ribosomal database using the short reads alignment tool SOAPnuke (v1.5.2) [58] and the aligned reads were removed (up to 5 mismatches were allowed). After removing reads with adaptor, N ratio containing greater than 10% and inferior quality, the filtered reads were aligned to the reference genome of rice (https://www.ncbi.nlm.nih.gov/assembly/GCF_001433935.1) using HISAT2 software (v2.0.4) [59] and re-assembled using StringTie (v1.0.4) [60]. To obtain the positional relationship of re-assembled transcripts, they were compared with known mRNAs and lncRNAs using cuffcompare, which was one of the tools of Cufflinks (v2.2.1) [61], and then the final transcripts were combined using Cuffmerge (one tool of Cufflinks, v2.2.1) [61].

### Identification of mRNAs and lncRNAs

LncRNAs was identified with reference to the study of Liu et al. [39]. The coding ability of combined transcripts (FPKM ≥0.5, Coverage > 1, Length > 200) were predicted using protein database Pfam (http://pfam.xfam.org/) [62] and three software, including Coding Potential Calculator (CPC, v0.9-r2, http:// CPC.cbi.pku.edu.cn) [63], Coding-Non-Coding Index (CNCI, https://github.com/www-bioinfo-org/CNCI) [64] and txCdsPredict (http://hgdownload.soe.ucsc.edu/admin/jksrc.zip). The four judgment methods are elaborated as follows: 1) If the transcripts were mapped to the Pfam database, they were recognized as mRNAs, otherwise lncRNAs; 2) CPC_threshold = 0, transcripts which have values greater than 0 were mRNAs, otherwise lncRNAs; 3) CNCI_threshold = 0, transcripts which have values greater than 0 were mRNAs, otherwise lncRNAs; 4) txCdsPredict_threshold = 500, transcripts which have values greater than 500 were mRNAs, otherwise lncRNAs. Transcripts were finally identified as mRNAs or lncRNAs when at least three of the four above methods were consistent.

### Differential expression analysis of lncRNAs

Clean reads were aligned to the reference genome using Bowtie2 software (v2.2.5) [65] and then the expression levels of transcripts were calculated using RSEM (v1.2.12) [66]. The normalized method used by RSEM software was FPKM, and the formula was as follows: FPKM = $\frac{10^6C}{NL/{10}^3}$ . In this formula, ‘C’ is the number of unique fragments for the target gene, ‘N’ is the total number of fragments which were uniquely matched the reference genome, and ‘L’ is the total number of bases in the coding region of the target gene. The calculated FPKM values, representing the gene expression levels, can be directly used to compare the gene expression differences among different samples. Correlations for three biological replications were calculated based on FPKM values using cor function in R (v3.3.0, https://www.r-project.org/). Software DEGseq [67] was used to analyze the difference of the comparison group. In this study, transcripts that exhibited fold change (FC) ≥ 2 (|log_2_FC| ≥ 1) and the false discovery rate (FDR) ≤ 0.001 were regarded as significantly differentially expressed transcripts. The differentially expressed lncRNAs were screened from the differentially expressed transcripts according to the ID of lncRNAs.

### Identification of lncRNAs target genes and GO analysis

LncRNAs regulate target genes in two ways, including *cis*- and *trans*-regulation. When lncRNA plays the *cis*-regulatory role, the location of lncRNA on the chromosome is close to the target gene, so the mRNA adjacent to lncRNA is selected as its target gene; when lncRNA plays the *trans*-regulatory role, it does not depend on the position relationship with the target gene, and its target gene can be predicted by calculating the binding energy. Specifically, according to the study of Liu et al. [39], target genes were analyzed in three steps: 1) the correlations between all identified lncRNAs and mRNAs were analyzed statistically (spearman values ≥0.6 and Pearson values ≥0.6); 2) lncRNAs were determined to play *cis*-regulatory roles when they located within 10 k upstream or 20 k downstream of the target genes; 3) when lncRNAs were not located in that range, RNAplex was used to analyze the binding energies of lncRNAs and mRNAs. If the binding energies were less than − 30, they were determined as lncRNAs with *trans*-regulatory effects. Furthermore, if lncRNA overlaps with the target gene, it will be further classified to 10 subclasses (such as lnc-overlap-mRNA and lnc-anti overlap-mRNA), which is conducive to increasing our understanding of the *cis*-regulated details of lncRNA [68, 69]. In addition, all identified potential target genes of differentially expressed lncRNAs (DE-lncRNAs) in all comparison groups were used for GO enrichment analysis using the WEGO website (http://wego.genomics.org.cn) with rice genome as the background.

### Prediction of lncRNA as the miRNA precursor

To predict lncRNAs that might be the precursors of the microRNAs (miRNAs), BLAST tool was used to align all lncRNAs to the miRbase (http://www.mirbase.org) [70]. The lncRNA was recognized as miRNA precursor when the coverage ratio of lncRNA sequence to the miRNA precursor sequence was over 90%, and the precursor sequences of miRNAs were from the previous data [2]. The secondary structures of lncRNA and miRNA precursor were plotted by RNAfold web server (http://rna.tbi.univie.ac.at/cgi-bin/RNAWebSuite/RNAfold.cgi). The interactive network relationships of lncRNA, miRNA and mRNA were displayed by Cytoscape software (v3.7.1, http://www.cytoscape.org).

### Validation of the data by qRT-PCR

To verify the accuracy of lncRNA sequencing data in this study, 9 lncRNAs and their predicted potential target genes were randomly selected from all expressed lncRNAs and verified by quantitative Real-Time PCR (qRT-PCR). Total RNAs of stems from five lines were extracted using TRIzol reagent (Invitrogen), and reverse transcribed with random primers. The lncRNA/mRNA specific primers were designed using Primer 5.0 software (http://www.premierbiosoft.com/index.html). The PCR amplifications were conducted using the SYBR@qPCR Mix (Toyobo) in the ABI Step One Plus Real-Time PCR System (Applied Biosystems, USA). The qRT-PCR reaction was processed as previously described with three biological replicates and three technical repeats [29]. In addition, *OsActin1* [71] was employed as internal control gene to normalize each lncRNA/mRNA threshold cycle reaction.

## Supplementary information

**Additional files 1: Figure S1.** Correlation coefficients for three biological replicates of gene expression data

**Additional files 2: Figure S2.** Differentially expressed lncRNAs in three progenies compared with their parents. FC stands for the fold change of DE-lncRNAs. The red number represents the number of up-regulated DE-lncRNAs and the blue number represents the number of down-regulated DE-lncRNAs

**Additional files 3: Figure S3.** The GO enrichment analysis of potential target mRNAs of lncRNAs with ELD expression patterns in L1710 (**A**), L1817 (**B**) and L1730 (**C**). Red mark ‘*’ indicated significantly enriched GO terms, of which the *P*-value was below 0.05

**Additional files 4: Table S1.** Summary of sequencing reads in fifteen libraries

**Additional files 5: Table S2.** The statistical analysis of assembled 16,038 novel transcripts

**Additional files 6: Table S3.** The normalized FPKM values for all lncRNAs and mRNAs in all samples

**Additional files 7: Table S4.** The regulatory relationship types of lncRNA-mRNA pairs

**Additional files 8: Table S5.** Targeting relationships between miRNAs and lncRNAs/mRNAs

**Additional files 9: Table S6.** Parental expression level dominance lncRNAs and its target mRNAs in three progenies

**Additional files 10: Table S7.** The primers used for quantitative real-time PCR

## Data Availability

The datasets analyzed during the current study are available in the NCBI’s Sequence Read Archive (SRA) repository (https://trace.ncbi.nlm.nih.gov/Traces/sra/sra.cgi?view=announcement) and the accession numbers of the fifteen runs are SRR9822767-SRR9822781. The web link of the reference genome of rice was https://www.ncbi.nlm.nih.gov/assembly/GCF_001433935.1. The web link of protein database Pfam was http://pfam.xfam.org/. The web link of miRbase was http://www.mirbase.org. All other data generated or analyzed during this study were included in this published article and the additional files.

## Abbreviations

ABA: Abscisic acid
BILs: Backcross introgression lines
CNCI: Coding-Non-Coding Index
CPC: Coding Potential Calculator
CTR1: CONSTITUTIVE TRIPLE RESPONSE1
DE-lncRNAs: Differentially expressed lncRNAs
*DEP1*: *DENSE AND ERECT PANICLE1*
DPA: Polyadenylation
ELD: Expression level dominance
FC: Fold change
FDR: False discovery rate
*FLC*: *FLOWERING LOCUS C*
GA: Gibberellin
KO: *Ent*-kaurene oxidase
lncRNA: Long non-coding RNA
miRNAs: micrornas
*OsTPP1*: *Trehalose-6-phosphate phosphatase*
*Phytochrome A*: *PHYA*
qRT-PCR: Quantitative Real-Time PCR
rRNAs: Ribosomal RNAs
*OsSPL3*: *SQUAMOSA PROMOTER-BINDING PROTEIN-LIKE3*
SRA: Sequence Read Archive
ss-RNAseq: Strand-specific RNA sequencing.
