# Supplementary figures and images for: Genome-wide identification and integrated analysis of lncRNAs in rice backcross introgression lines (BC2F12)
Source: BMC Plant Biol. 2020 Jun 29;20:300. doi: 10.1186/s12870-020-02508-y (PMC7325253; doi:10.1186/s12870-020-02508-y)

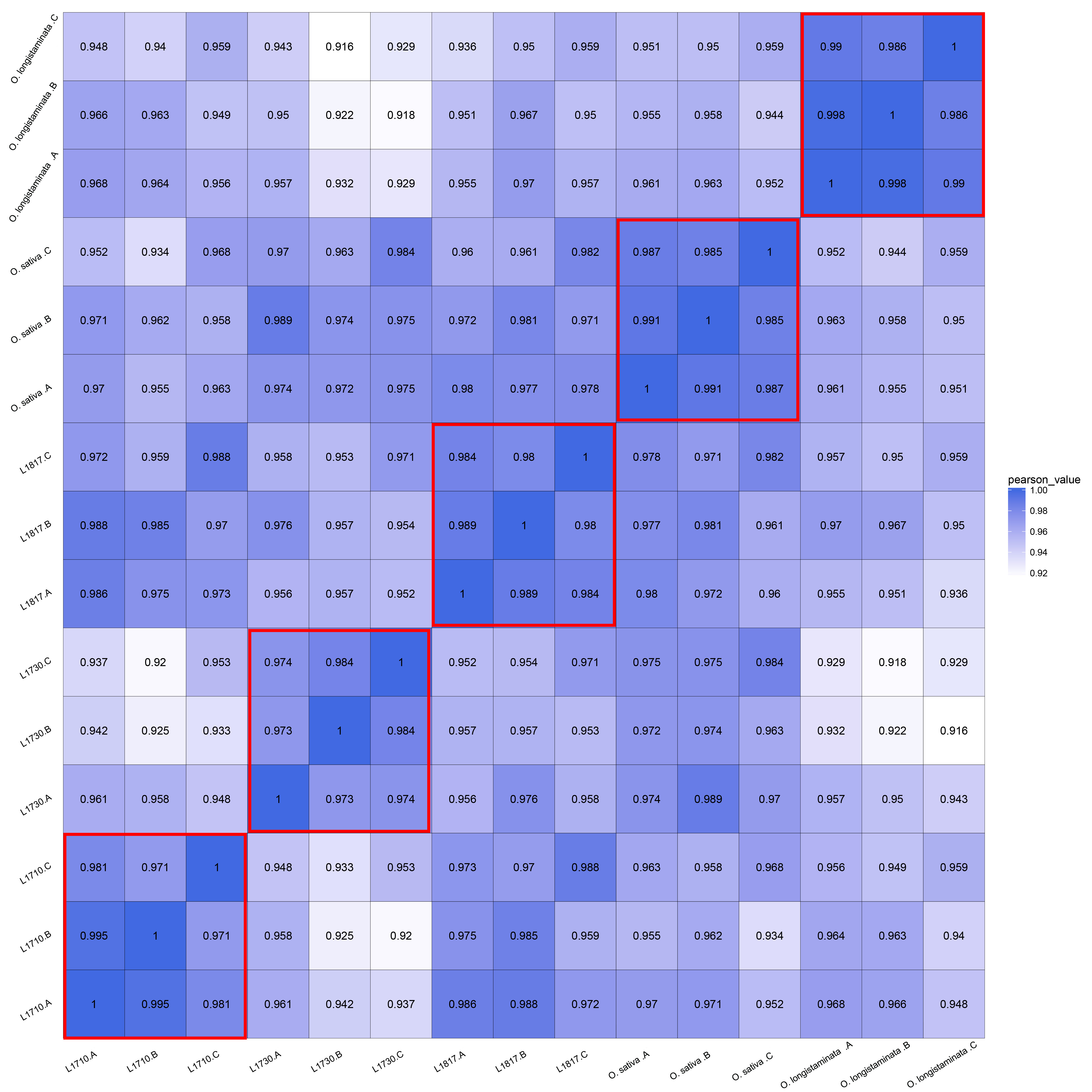

Supplement: Supplementary file 1 — Additional files 1: Figure S1. Correlation coefficients for three biological replicates of gene expression data [file 12870_2020_2508_MOESM1_ESM.jpg]

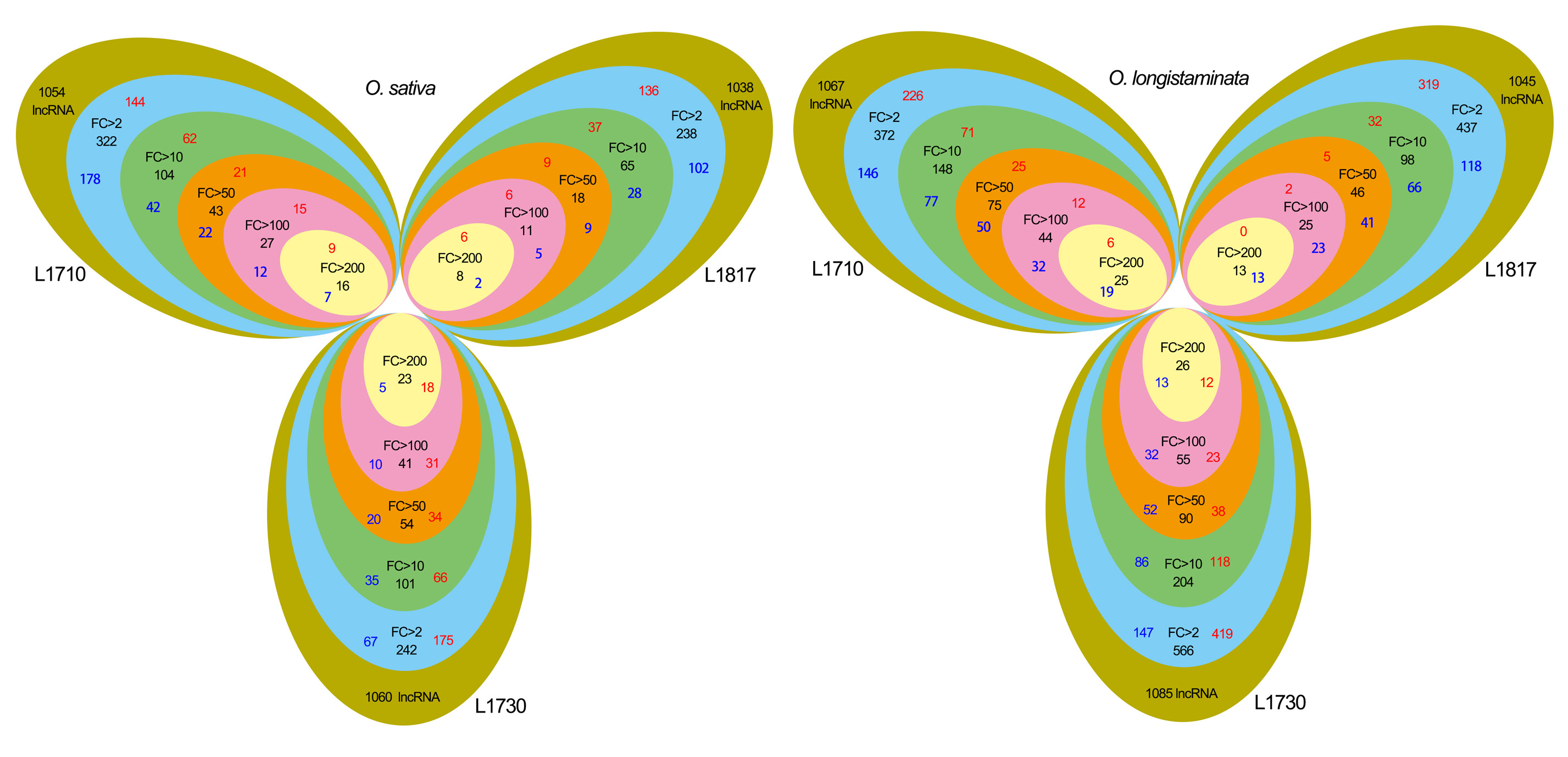

Supplement: Supplementary file 2 — Additional files 2: Figure S2. Differentially expressed lncRNAs in three progenies compared with their parents. FC stands for the fold change of DE-lncRNAs. The red number represents the number of up-regulated DE-lncRNAs and the blue number represents the number of down-regulated DE-lncRNAs [file 12870_2020_2508_MOESM2_ESM.jpg]

a


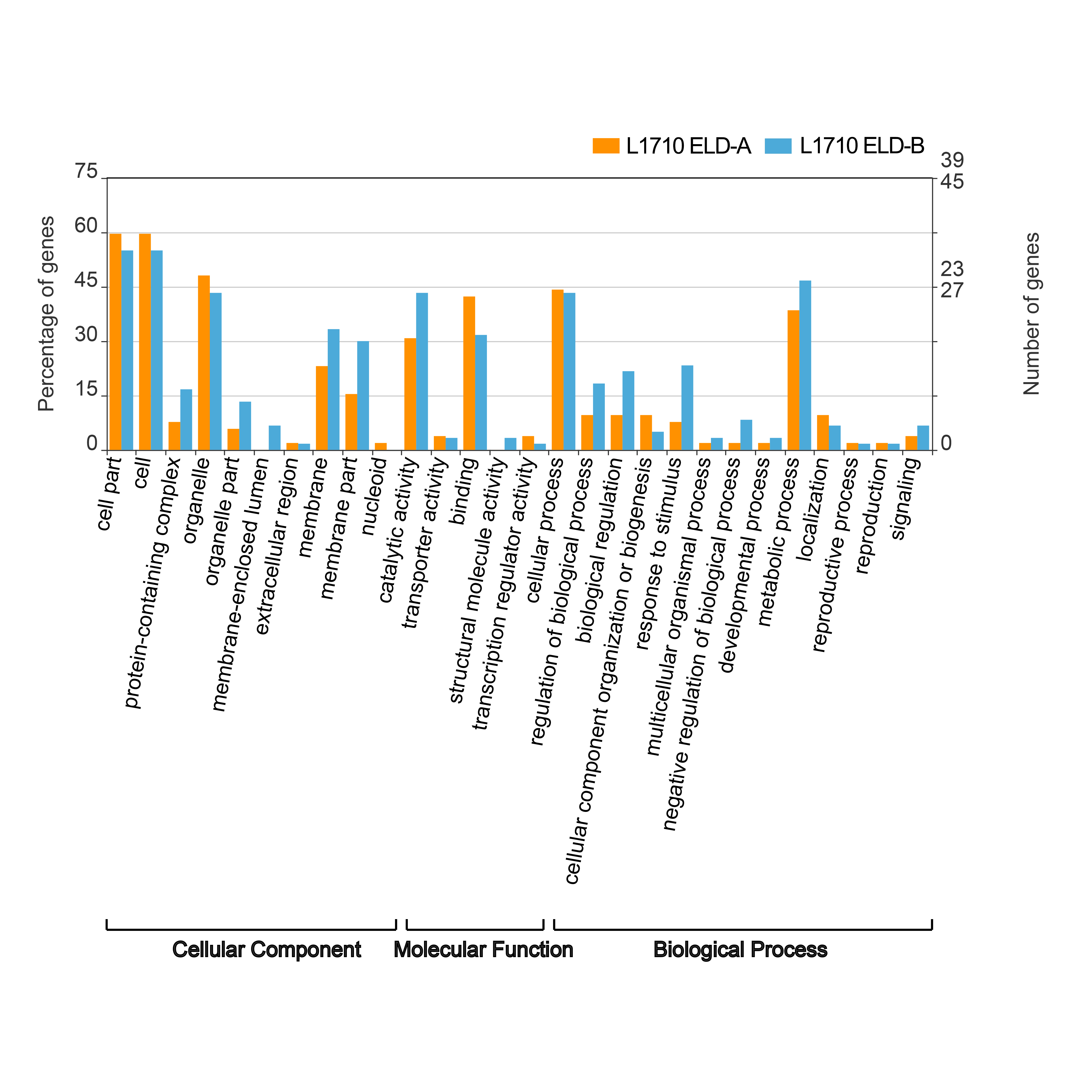


b


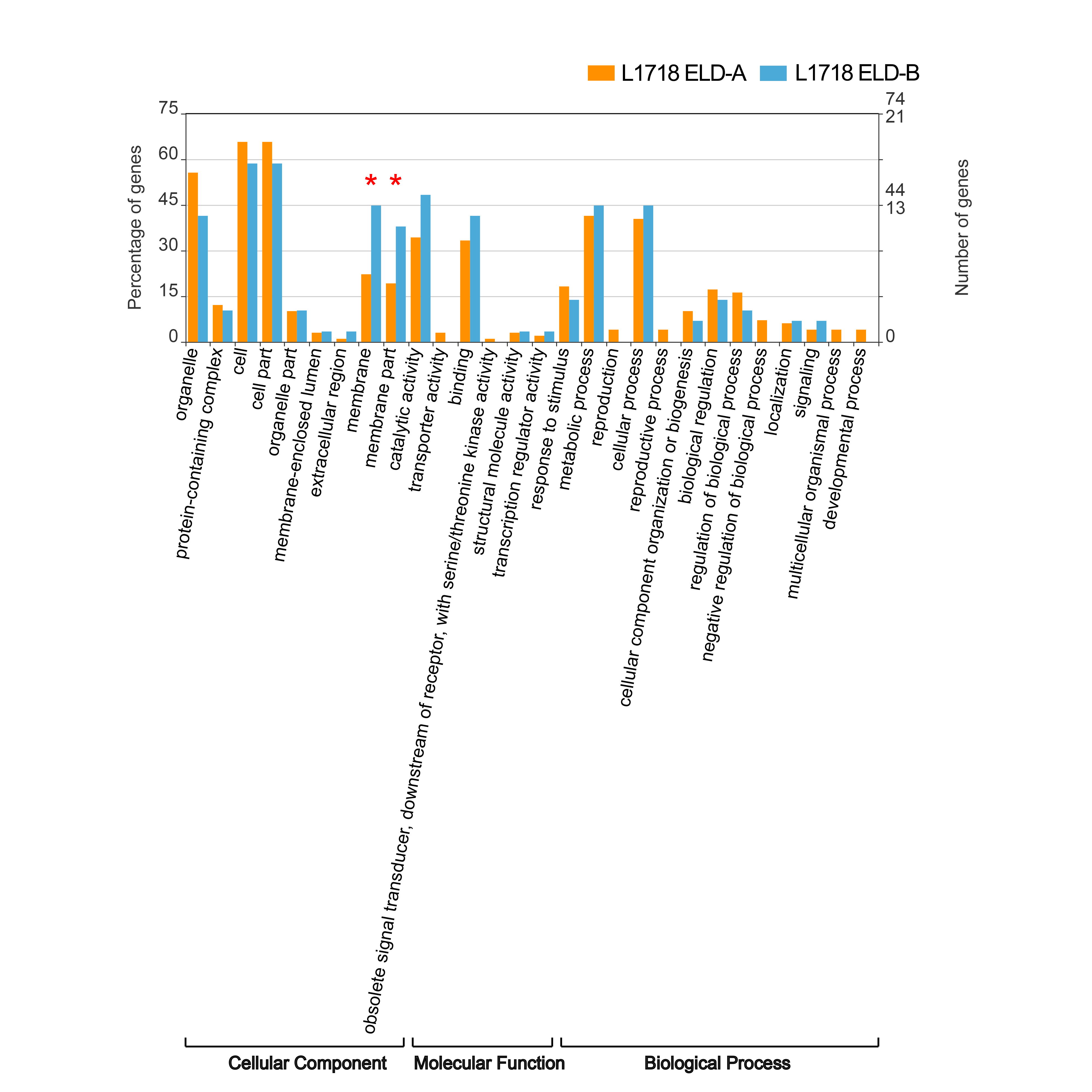


c


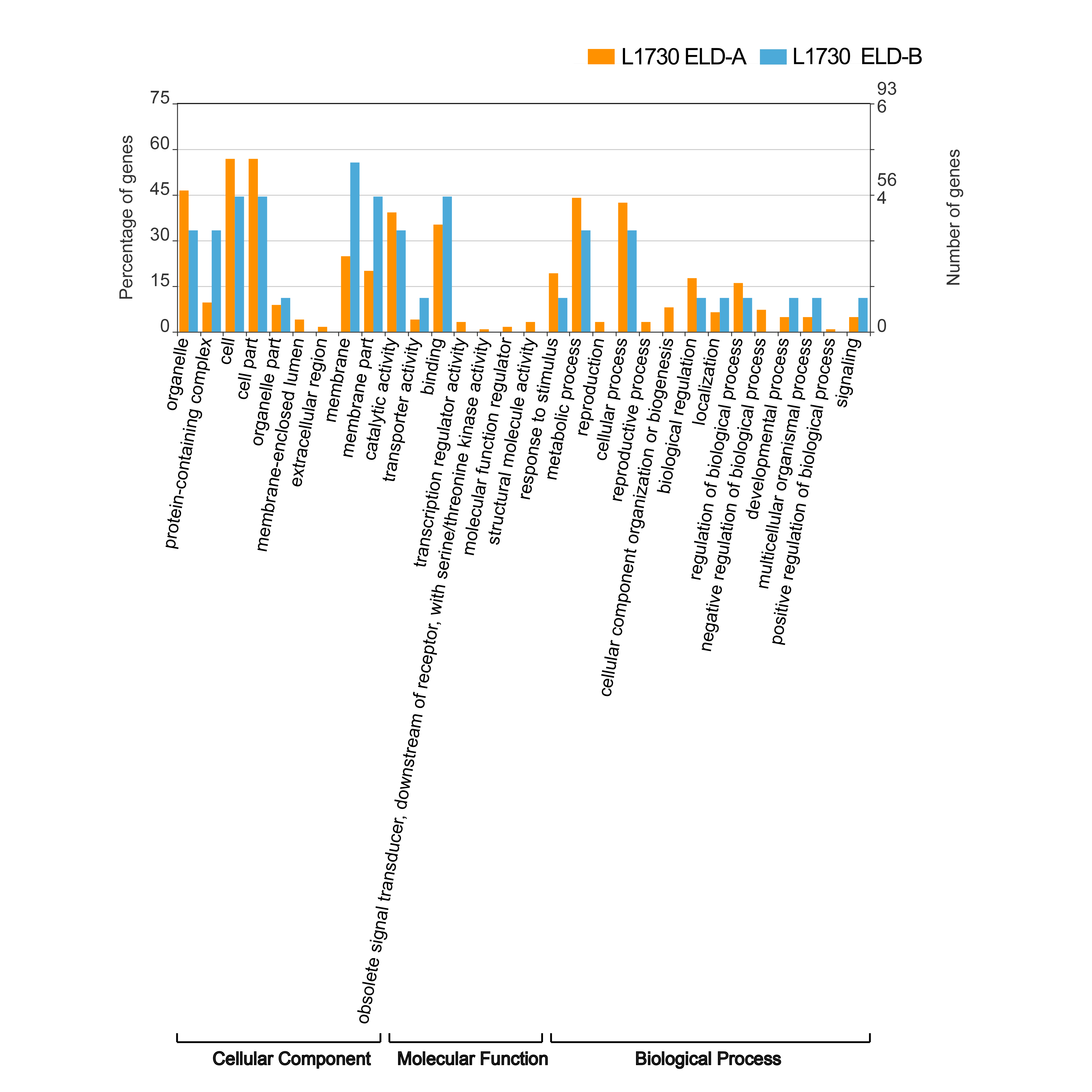

Supplement: Supplementary file 3 — Additional files 3: Figure S3. The GO enrichment analysis of potential target mRNAs of lncRNAs with ELD expression patterns in L1710 (A), L1817 (B) and L1730 (C). Red mark ‘*’ indicated significantly enriched GO terms, of which the P-value was below 0.05 [file 12870_2020_2508_MOESM3_ESM.docx]
